# Schwann-Spheres Derived from Injured Peripheral Nerves in Adult Mice - Their *In Vitro* Characterization and Therapeutic Potential

**DOI:** 10.1371/journal.pone.0021497

**Published:** 2011-06-24

**Authors:** Takehiko Takagi, Ken Ishii, Shinsuke Shibata, Akimasa Yasuda, Momoka Sato, Narihito Nagoshi, Harukazu Saito, Hirotaka J. Okano, Yoshiaki Toyama, Hideyuki Okano, Masaya Nakamura

**Affiliations:** 1 Department of Orthopaedic Surgery, Keio University School of Medicine, Tokyo, Japan; 2 Department of Physiology, Keio University School of Medicine, Tokyo, Japan; 3 Center for Integrated Medical Research, Keio University, Tokyo, Japan; 4 Department of Orthopaedic Surgery, Murayama Medical Center, National Hospital Organization, Tokyo, Japan; Centre National de la Recherche Scientifique, University of Bordeaux, France

## Abstract

Multipotent somatic stem cells have been identified in various adult tissues. However, the stem/progenitor cells of the peripheral nerves have been isolated only from fetal tissues. Here, we isolated Schwann-cell precursors/immature Schwann cells from the injured peripheral nerves of adult mice using a floating culture technique that we call “Schwann-spheres." The Schwann-spheres were derived from de-differentiated mature Schwann cells harvested 24 hours to 6 weeks after peripheral nerve injury. They had extensive self-renewal and differentiation capabilities. They strongly expressed the immature-Schwann-cell marker p75, and differentiated only into the Schwann-cell lineage. The spheres showed enhanced myelin formation and neurite growth compared to mature Schwann cells *in vitro*. Mature Schwann cells have been considered a promising candidate for cell-transplantation therapies to repair the damaged nervous system, whereas these “Schwann-spheres" would provide a more potential autologous cell source for such transplantation.

## Introduction

In recent years, multipotent somatic stem cells have been identified in various adult tissues. In the peripheral nerves, stem/progenitor cells that are self-renewing and multipotent, with the potential to differentiate into neurons, glial cells, and myofibroblast, have been detected and isolated from fetal [1], but not adult tissues.

After peripheral nerve injury, mature Schwann cells undergo a reversion in their molecular phenotype, and come to resemble those observed in fetal immature nerves [2]. However, no report has explored how far these cells dedifferentiate, even though recent progress in understanding neural-crest and Schwann-cell development has revealed a rather complete picture of glial development in the early peripheral nerves [3]. In the present study, we sought to determine whether mature Schwann cells in adult peripheral nerves that dedifferentiate into stem/progenitor cells after injury could form spheres in floating culture conditions, even though such spheres cannot be obtained by culturing the dissociated cells of intact peripheral nerves from neonates [4] or adult mice [5].

Here, we cultured the dedifferentiated Schwann cells obtained from the injured peripheral nerves of adult mice at the specific time-point under the floating culture condition and isolated Schwann-cell precursors/immature Schwann cells, as spheres, which we called “Schwann-spheres." This is the first report showing that “Schwann-spheres" can be obtained from adult peripheral nerves. Moreover, their differentiation, myelination, and neurite growth promoting properties *in vitro* suggested their potential use in cell transplantation therapy for the damaged nervous system.

## Materials and Methods

### Animals and surgical procedures

Normal, specific pathogen-free, adult C57BL/6J mice were purchased from CLEA Japan, Inc., Tokyo, Japan. Nestin-EGFP transgenic mice carry enhanced green fluorescent protein (EGFP) under the control of the second intronic enhancer of the nestin gene, which acts selectively in stem/progenitor cells [6]. Transgenic mice expressing Cre recombinase under control of the MBP promoter (MBP-Cre)[7] were mated with EGFP reporter mice (CAG-CAT*^loxP/loxP^*-EGFP) [8] to obtain MBP-Cre/Floxed-CAG-EGFP double-transgenic mice [9].

The adult C57BL/6J mice, Nestin-EGFP mice, and MBP-Cre/Floxed-CAG-EGFP mice (female, 7-8 weeks old) were anesthetized using an intraperitoneal injection of ketamine (100 mg/kg) and xylazine (10 mg/kg). The animals were housed in groups under a 12-hour light/dark cycle, with access to food and water ad libitum. The sciatic nerve was exposed through a dorsal gluteal muscle-splitting approach. The nerve was subjected to a contusive injury at the sciatic notch using a brain aneurysm clip (Sugita clip; Mizuho Ikakogyo, Tokyo, Japan). The clip was closed and left in place for 5 min with a holding force of approximately 50 g. All interventions and animal care procedures were performed in accordance with the Laboratory Animal Welfare Act, the Guide for the Care and Use of Laboratory Animals (National Institutes of Health), and the Guidelines and Policies for Animal Surgery provided by the Animal Study Committee of Keio University, and were approved by the Ethics Committee of Keio University.

### Sphere-forming cultures

Cells were dissociated from the distal portion of sciatic nerves before the injury and at 6 or 24 hours, 3, 7, or 10 days, or 2, 3, or 6 weeks after the injury. The cells (1×10^5^ cells/ml) were transferred to a sphere-forming floating medium consisting of DMEM/F-12 (1:1) (Gibco, Carlsbad, CA, USA) supplemented with insulin (25 mg/ml), transferrin (100 mg/ml), progesterone (20 nM), sodium selenate (30 nM), putrescine (60 nM) (all from Sigma-Aldrich, St. Louis, USA), recombinant human EGF (100 ng/ml) (Pepro Tech, Rocky Hill, NJ, USA), human FGF-basic (100 ng/ml) (Pepro Tech), B27 (20 ng/ml) (modified from Nagoshi et al. [10]), and 10% FBS (Equitech-Bio, Kerrville, TX, USA). By comparison, neural crest stem cells (NCSCs) were dissociated from the E14.5 dorsal root ganglion (DRG) and neural stem cells (NSCs) were dissociated from the E14.5 striatum [11]. The NCSCs and NSCs were transferred to the sphere-forming floating medium without FBS. The cells were cultured in an incubator at 37°C for 7 days. For clonal sphere expansion, the cells were cultured in the above medium with 0.8% methylcellulose (Nacalai Tesque, Kyoto, Japan) [12].

For the secondary sphere formation assays, the 7-day primary spheres were collected, incubated in 0.25% trypsin-EDTA for 30 min at 37°C, and triturated until a single-cell suspension was obtained. The cells were centrifuged at 800 rpm for 5 min at 4°C, and resuspended in the aforementioned sphere culture medium. The NCSCs and NSCs were resusupended in the sphere culture medium without FBS.

### Characterization of spheres derived from injured peripheral nerves

#### Assays using nestin-EGFP and MBP-Cre/Floxed-EGFP mice

Spheres obtained from the contused sciatic nerves of 7-8-week-old adult Nestin-EGFP mice and MBP-Cre/Floxed-EGFP mice were verified by direct EGFP-fluorescent imaging to be Nestin-positive and derived from MBP-positive cells.

#### Immunocytochemistry

Spheres were postfixed for 6 hours in 4% paraformaldehyde (PFA), soaked overnight in 10% sucrose followed by 30% sucrose, and embedded in cryomolds for sectioning at 6–8 µm. The spheres were immunostained using the anti-undifferentiated-cell marker, Nestin (mouse IgG1, 1:200, BD Pharmingen, San Jose, CA); Schwann-cell markers, P0 (PZO; chick IgG, 1:200, Aves Labs, Tigard, OR, USA) and p75 (rabbit IgG, 1:200, Chemicon, Billerica, MA, USA), or the proliferative-cell marker, PCNA (rabbit IgG, 1:500, Oncogene Research Product, La Jolla, CA, USA), to define the cell population. Immunoreactivity was visualized using secondary antibodies conjugated with Alexa 488 or Alexa 555 (Molecular Probes). Nuclear counterstaining was performed with Hoechst 33342 (10 mg/ml, Sigma, St. Louis, MO, USA). The samples were observed with a universal fluorescence microscope (AxioImager M1; Carl Zeiss, Jena, Germany).

#### Differentiation analysis

Spheres were plated on poly-L-lysine (PLL) (Sigma)-coated 8-well chamber slides (Iwaki, Tokyo, Japan) and cultured for 7 days in the following differentiation medium: DMEM/F12 (1∶1) supplemented with 10% FBS, without any growth factors [10]. For immunocytochemistry, the cells were fixed in 4% PFA and stained with the following primary antibodies: anti-p75 (rabbit IgG, 1∶200, Chemicon) and anti-P0 (PZO; chick IgG, 1∶200, Aves Labs), to examine their differentiation into the mature stage of the Schwann-cell lineage. Secondary antibodies were anti-rabbit IgG (Alexa 488) and anti-chick IgG (Alexa 568).

The distal portions of intact or injured sciatic nerves (day 7 after the injury) from MBP-Cre/Floxed-EGFP mice were postfixed for 24 hours in 4% PFA, soaked overnight in 10% sucrose followed by 30% sucrose, and embedded in a cryomold for sectioning at 10 µm. The primary antibodies were anti-GFP (goat IgG, 1∶200, Rockland, Gilbertsville, PA) and anti-p75 (rabbit IgG, 1∶200, Chemicon). The secondary antibodies were anti-goat IgG (Alexa 488) and anti-rabbit IgG (Alexa 555). Nuclear counterstaining was performed with Hoechst 33342 (10 mg/ml, Sigma). The samples were observed with a confocal laser scanning microscope (LSM510, Carl Zeiss).

To examine the tri-lineage differentiation potential, spheres derived from injured adult sciatic nerves from MBP-Cre/Floxed-CAG-EGFP mice were plated on PLL-coated 8-well chamber slides and cultured for 7 days in the above-described differentiation medium. For immunocytochemistry, the cells were fixed in 4% PFA and stained with the following primary antibodies: anti-GFP (goat IgG, 1∶200, Santa Cruz Biotechnology, Santa Cruz, CA, USA), anti-P0 (PZO; chick IgG, 1∶200, Aves Labs), anti-S100 (rabbit IgG, 1∶500, Dako, Glostrup, Denmark), anti-β-III tubulin (mouse IgG2b, 1∶500, Sigma), and anti-αSMA (mouse IgG2a, 1∶1000, Sigma). Secondary antibodies were the following: anti-goat IgG (Alexa 488), anti-rabbit IgG (Alexa 555), anti-chick IgG (Alexa 568), anti-mouse IgG2b (Alexa 488), and anti-mouse IgG2a (Alexa 647). The samples were observed with a universal fluorescence microscope (AxioImager M1; Carl Zeiss).

#### RT-PCR assay

Total RNA was isolated from each sample with Trizol reagent (Invitrogen, Carlsbad, CA, USA) and DNase I treatment. Total RNA (1 µg) was used to synthesize cDNA with oligo-d(T) primers. The cDNA synthesis was performed at 42°C for 50 min in a final volume of 20 µl, according to the manufacturer's instructions for Superscript III reverse transcriptase (Invitrogen). To normalize the template cDNA, the ubiquitously expressed *β-actin* mRNA was used as a reference. PCR was performed with KOD plus DNA polymerase (Toyobo, Osaka, Japan) according to the manufacturer's instructions. The PCR products were resolved by electrophoresis in 1-3% agarose gels, and the bands were visualized with ethidium bromide under UV light [10]. The PCR products were confirmed by sequencing. The primers are listed in Table 1.

**Table 1:** Primer Sequences.

	Temp.	Size	sense	antisense
*Nestin*	60	212	CAGCTGAGCCTATAGTTCAACGC	GAAACAAGATCTCAGCAGGCTGAG
*Musashi1*	60	542	GGCTTCGTCACTTTCATGGACC	GGGAACTGGTAGGTGTAACCAG
*Pax3*	60	171	AACAAGCTGGAGCCAATCAACTG	CTGAGGTCTGTGGACGGTGCTA
*Sox9*	60	263	CACGGAACAGACTCACATCTC	TGCTCAGTTCACCGATGTCCA
*Sox10*	60	134	ACGCACTGAGGACAGCTTTGA	ATGAGGTTATTGACACGGAACTGG
*p75*	60	115	GAGTGCTGCAAAGCCTGCAA	TGGCGCTCACCACGTCAGAG
*β-actin*	60	131	TGACAGGATGCAGAAGGAGA	GCTGGAAGGTGGACAGTGAG

### Myelination and neurite-extension assays

DRG neurons were co-cultured with cells derived from intact sciatic nerves or Schwann-spheres using the modified method of Hoshikawa et al [13]. The DRGs were taken from adult mice, dissociated with collagenase and trypsin, and seeded on 8-well chamber slides coated with poly-L-lysine at 200,000 cells per well. Thereafter, 250,000 cells from the spheres or intact nerves were seeded onto the DRG cultures in DMEM/F12 medium. The cocultures were incubated for 2 weeks, and then anti-MBP and anti-βIII-tubulin antibodies were applied, followed by the appropriate secondary antibodies. The proportions of MBP-positive cells (MBP-positive cells/total cells) were counted and the lengths of the longest βIII-tubulin-positive neurite were measured in a 0.6 mm^2^ field by surveying six fields. The average was calculated.

### Statistical analysis

All values are presented as the mean ± standard error of the mean (SEM). Statistical significance was determined as *p*<0.05 using one-factor ANOVA and the Tukey-Kramer test for the primary and secondary sphere-forming assays. Student's *t-*test was used to compare the data between groups for the myelination and neurite outgrowth assays.

## Results

### Adult injured sciatic nerves include sphere-forming cells with a high self-renewal capability

**Figure 1:**
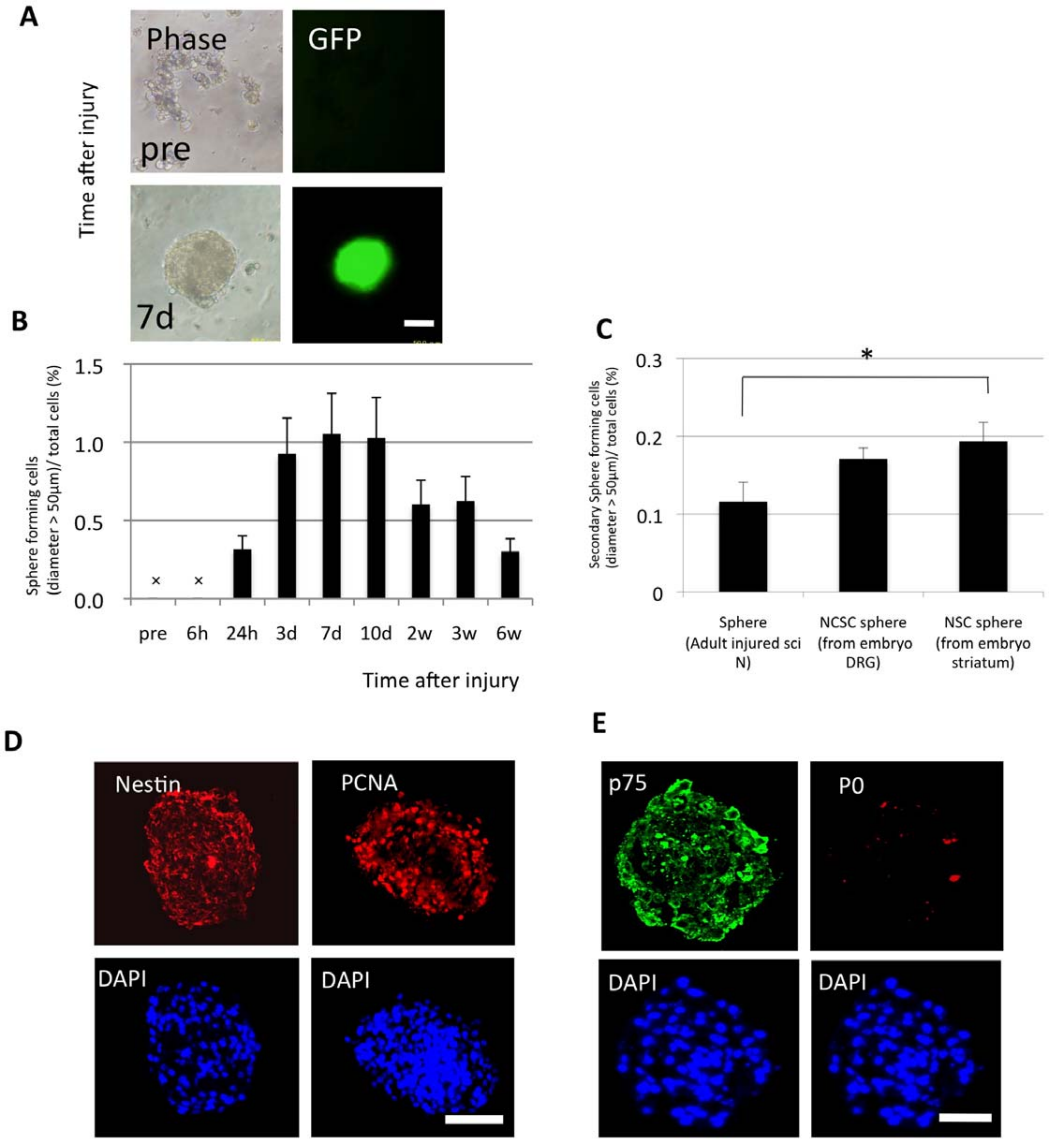
Sphere-forming capacity of cells derived from the injured sciatic nerves of adult mice. (A) Phase-contrast and direct EGFP-fluorescent images showing the spheres formed from the injured nerves of adult *nestin*-EGFP mice were EGFP+ after 7 days in floating culture (pre, pre-injured; 7d, 7 days after injury; Scale bar represents 50 µm). (B) Percentage of sphere-forming cells derived from intact and injured sciatic nerves at 6 or 24 hours, 3, 7, or 10 days, or 2, 3, or 6 weeks after injury (mean ± SEM; n = 6 per group; x, no spheres observed). While the sciatic nerves harvested 24 hours to 6 weeks after injury could form spheres under floating culture conditions, those harvested at the other time points did not. (C) Secondary sphere-forming capability of the spheres derived from adult sciatic nerves compared to those derived from neural-crest stem cells (from fetal DRGs) and neural stem cells (from fetal striatum). Spheres from injured adult sciatic nerve could be passaged and formed secondary spheres, indicating their self-renewing ability. One-factor ANOVA and the Tukey-Kramer test were applied (mean ± SEM; n =  per group; *p<0.05). Scale bar represents 50 µm. (D) The images of immunostained spheres contain Nestin- and PCNA-positive cells. (E) Most of the cells expressed the undifferentiated Schwann cell marker, p75, whereas few cells were positive for myelinating Schwann cell marker, P0. Scale bar represents 50 µm.

To determine whether there were sphere-initiating cells within adult intact and injured peripheral nerves, we cultured cells derived from the intact and injured sciatic nerves of adult *nestin*-EGFP mice [6]. We succeeded in obtaining spheres from the injured sciatic nerves in floating culture, when the nerves were harvested at certain time points after the injury. In contrast, no spheres were obtained from intact sciatic nerves. These spheres from *nestin*-EGFP mice were positive for EGFP, suggesting that they were Nestin-positive immature cells (Fig. 1**A**). The distal part of the sciatic nerves harvested 24 hours to 6 weeks after crush injury, but not at other time points, could successfully generate spheres in floating culture. The sphere-forming capacity peaked in the samples derived from nerves harvested 3 to 10 days after the injury. Quantitative analysis indicated that approximately 1% of all the viable cells were sphere-initiating cells during this time (Fig. 1**B**).

To assess the self-renewing capacity of these spheres, secondary sphere-forming assays were performed. Primary spheres derived from sciatic nerves harvested 7 days after injury were dissociated into single cells and cultured again in the sphere-forming floating medium. As positive controls, the primary spheres of neural crest stem cells obtained from fetal DRG and neural stem cells from fetal striatum were also dissociated and replated to form secondary spheres. Secondary spheres were successfully obtained from the dissociated primary sciatic-nerve-derived spheres, although the sphere-forming rate was lower than the neural crest stem cells and neural stem cells (Fig. 1**C**).

To characterize the sphere-forming cells derived from injured adult sciatic nerves, frozen sections of the spheres were stained with various cell markers. The spheres were positive for both the undifferentiated-cell marker Nestin [14] and the proliferative-cell marker PCNA (Fig. 1**D**). Interestingly, most of the cells in the spheres expressed undifferentiated-Schwann-cell marker, p75, and a small proportion of them were positive for the myelinating-Schwann-cell marker, P0 (Fig. 1**E**). These results suggested that these spheres consisted of Schwann-cell precursors/immature Schwann cells; we therefore called them, “Schwann-spheres."

### Characterization of the Schwann-spheres derived from adult injured sciatic nerve

**Figure 2:**
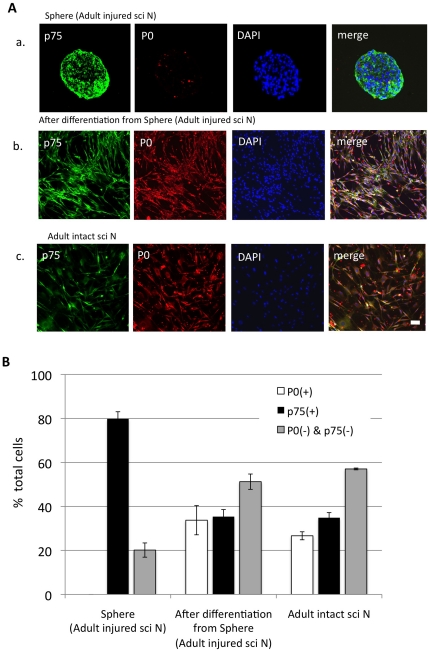
Differentiation assay of the Schwann-spheres derived from injured adult sciatic nerve. Immunostaining (A) with p75, a marker for immature and non-myelinating Schwann cells, and P0, a marker for myelinating Schwann cells, and quantitative analysis (B) (a, Sphere from injured adult sciatic nerves; b, Sphere after differentiation; c, Schwann cells from intact sciatic nerve; mean ± SEM; n =  per group). After inducing their differentiation, most Schwann-sphere cells differentiated into P0-positive mature Schwann cells, which closely resembled the Schwann cells from intact sciatic nerves. Scale bar represents 100 µm.

The majority of the cells in the Schwann-spheres were positive for p75, a marker for immature and non-myelinating Schwann cells, whereas very few cells were positive for P0, a marker for myelinating Schwann cells (Fig. 2**A** **–a and B–a**). We next asked whether the Schwann-spheres could differentiate into mature Schwann cells *in vitro*. After being cultured for 7 days in differentiation medium [10], approximately 37% of the total cells had differentiated into P0-positive mature Schwann cells (Fig. 2**A** **–b and B–b**), which had a very similar morphology to the mature Schwann cells derived from adult intact sciatic nerves (Fig. 2**A** **–c and B–c**).

**Figure 3:**
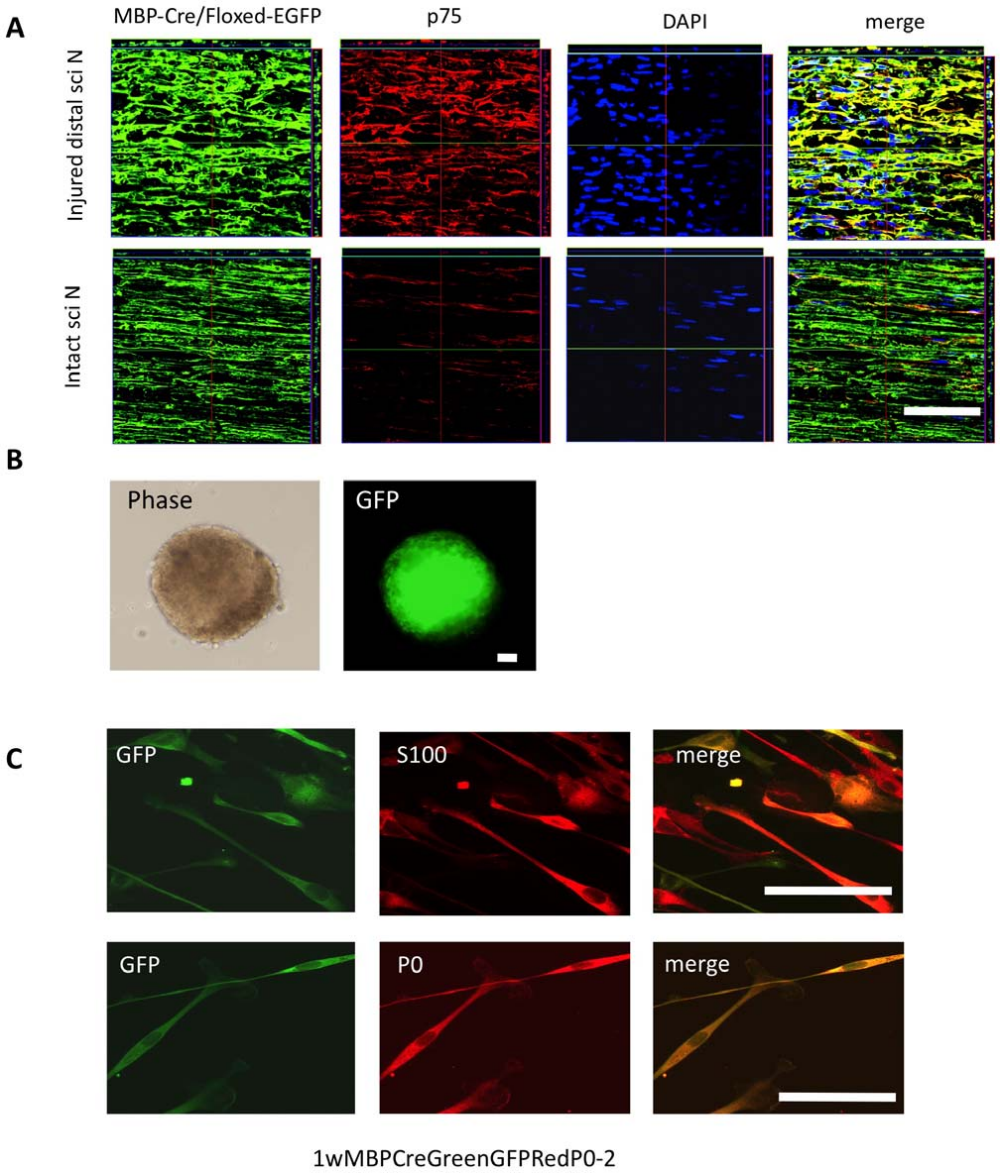
Expression pattern of EGFP in the injured sciatic nerves of adult MBP-Cre/Floxed-EGFP mice. (A) Double immunostaining for GFP and p75 revealed that most of the GFP-positive cells derived from the injured distal sciatic nerves were positive for p75, indicating that the myelinating mature Schwann cells could de-differentiate to an immature stage after peripheral nerve injury. (B) Phase-contrast and direct EGFP-fluorescent images showing that the spheres formed from EGFP+ cells after 7 days in floating culture of the injured peripheral nerves from adult MBP-Cre/Floxed-EGFP mice. (C) Trilineage differentiation potential of the spheres derived from the injured adult sciatic nerves of MBP-Cre/Floxed-EGFP mice. Double immunocytochemistry revealed that the EGFP+ cells were positive for glial-cell markers, S100 and P0. The EGFP+ spheres derived from the injured adult sciatic nerves differentiated into glial cells, but not into neurons or myofibroblasts (Fig. S1). Scale bar represents 50 µm.

Furthermore, to determine the origin of the Schwann-spheres, we induced a contusive sciatic nerve injury in MBP-Cre/Floxed-EGFP mice. In these transgenic mice, transient activation of the MBP promoter induces Cre-mediated recombination, indelibly tagging the MBP-positive mature Schwann cells with EGFP expression [7], [8], [9]. Double immunostaining for GFP and p75 in frozen sections of the distal part of the injured sciatic nerves revealed that most of the GFP-positive cells were positive for p75, whereas very few of the GFP-positive cells in intact sciatic nerves were p75-positive (Fig. 3**A**), suggesting that myelinating mature Schwann cells could de-differentiate to the immature stage after peripheral nerve injury. These EGFP-positive cells could form spheres under floating culture conditions (Fig. 3**B**), whereas EGFP-negative cells did not (data not shown). These findings suggested that the spheres were originally derived from MBP-positive mature Schwann cells in the pre-injury sciatic nerves, and that the spheres contained Nestin-positive immature cells (Fig. 1**A**). We also examined the trilineage differentiation potential of the spheres derived from the injured adult sciatic nerves of MBP-Cre/Floxed-EGFP mice. The EGFP+ spheres derived from these injured adult sciatic nerves differentiated into glial cells (Fig. 3**C**), but not into neurons or myofibroblasts (Fig. S1). These spheres could differentiate only into the Schwann-cell lineage, suggesting that mature Schwann cells de-differentiate into Schwann-cell precursors/immature Schwann cells, but not into neural-crest stem cells after injury.

### Schwann-spheres derived from injured sciatic nerves strongly express immature-Schwann-cell markers

**Figure 4:**
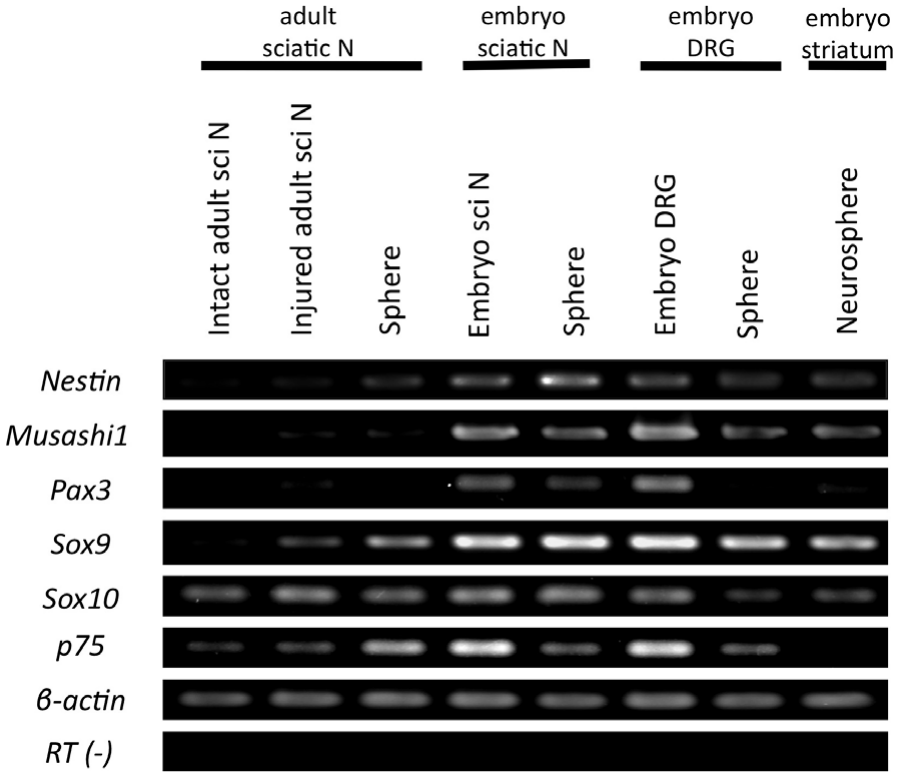
RT-PCR was conducted to evaluate the mRNA expression of various stem-cell and Schwann-cell markers. The injured adult sciatic nerve-derived spheres showed a higher expression of the immature-cell markers *Nestin* and *Musashi-1* and neural-crest markers *Pax3* and *Sox9* than the intact and injured adult sciatic nerves. However, their expression of these genes was lower than that of spheres derived from fetal sciatic nerves and DRGs. The intact and injured adult sciatic nerves, fetal sciatic nerves, DRGs, and striatum expressed *Sox10*. The expression of *p75* was observed in the spheres derived from injured adult sciatic nerves, fetal sciatic nerves, and DRGs.

Reverse transcription-polymerase chain reaction (RT-PCR) analysis was conducted to evaluate the mRNA expression of various stem-cell and Schwann-cell markers in the injured adult sciatic nerve-derived spheres and fetal neural crest-derived spheres (Fig. 4). The spheres derived from injured adult sciatic nerves showed higher expression of the immature-neural-precursor cell markers *Nestin* and *Musashi-1* than were seen in the intact and injured adult sciatic nerves. The neural-crest markers *Pax3* and *Sox9* were also expressed in the injured adult sciatic nerves and Schwann-spheres. However, their expression of these genes was lower than that of spheres derived from fetal sciatic nerves or DRGs. Intact and injured adult sciatic nerves, fetal sciatic nerves, DRGs, and striatum all expressed *Sox10* as expected, since this gene is expressed at all stages of the Schwann-cell lineage [15] and is deeply involved in the development of the central nervous system [16]. The expression of *p75,* the marker of immature and non-myelinating Schwann cells, was observed in the adult sciatic-nerve-derived Schwann-spheres, as well in fetal sciatic-nerve- and DRG-derived spheres. Interestingly, the *p75* expression in the cells from the injured adult sciatic nerve increased after sphere formation, but decreased in the fetal sciatic nerve- and DRG-derived spheres.

### Schwann-spheres show higher potentials for myelination and neurite outgrowth than do mature Schwann cells *in vitro*

**Figure 5:**
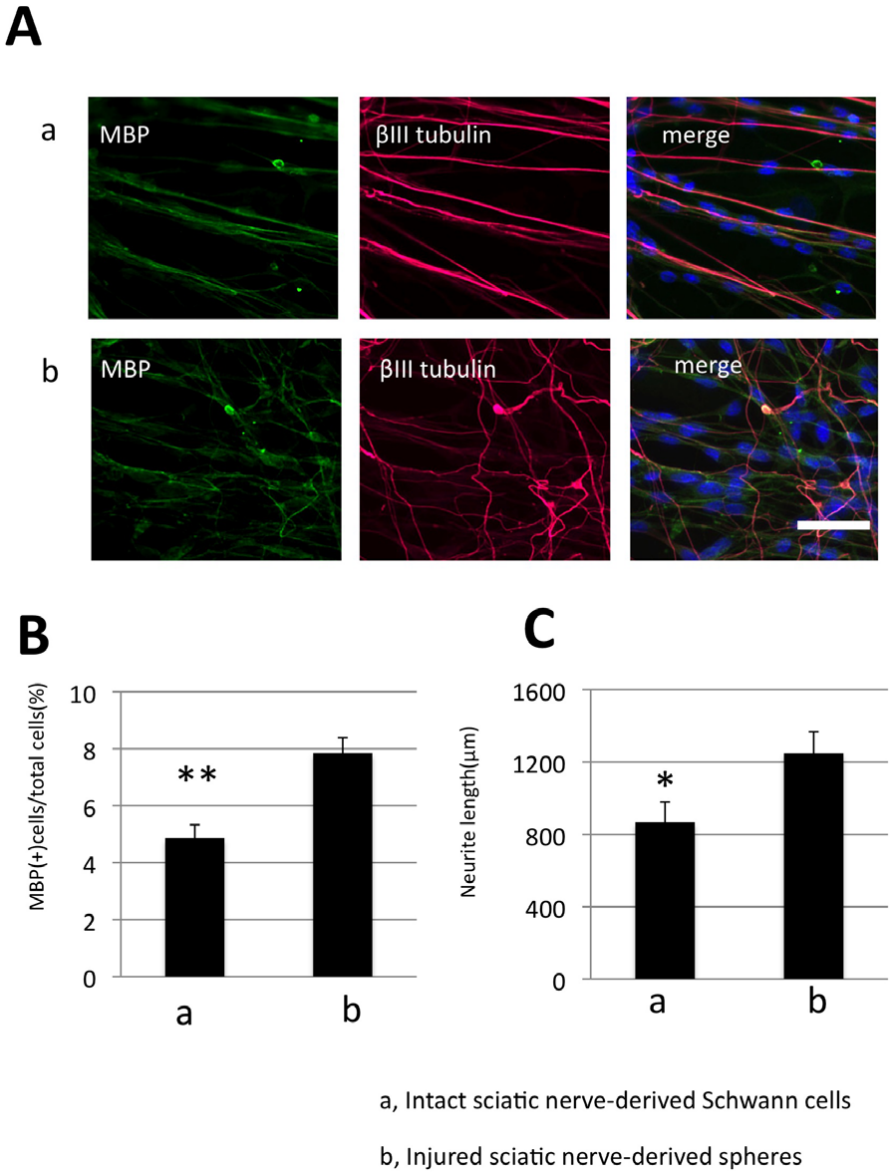
Myelination and neurite-growth assays. DRG neurons were co-cultured with mature Schwann cells from intact sciatic nerves or with Schwann-spheres derived from sciatic nerves harvested 1 week after injury. The cells were then double-stained for MBP and βIII-tubulin (A). The proportion of MBP-positive cells (MBP-positive cells/total cells) (B, myelination assay) and length of the longest βIII-tubulin-positive neurites (C, neurite outgrowth assay) in a 0.6-mm^2^ field were measured. Schwann-spheres caused enhanced myelination and neurite outgrowth compared with mature Schwann cells derived from intact sciatic nerves. Student's t-test was used to compare the data between groups (mean ± SEM; n =  per group; *, p<0.05; **, p<0.01). Scale bar represents 50 µm.

To examine the Schwann-spheres' therapeutic potential, we performed myelination and neurite growth assays *in vitro* [17]. DRG neurons were co-cultured with mature Schwann cells or with Schwann-spheres derived from injured adult sciatic nerves, and stained for MBP and βIII-tubulin (Fig. 5**A**). Both the number of MBP-positive myelin-forming Schwann cells in myelination assay [13] (Fig. 5**B**) and the length of the βIII-tubulin-positive neuritis in neurite outgrowth assay; [18] (Fig. 5**C**) were significantly greater in the co-culture with the Schwann-spheres derived from injured sciatic nerve compared with the co-culture with mature Schwann cells derived from intact sciatic nerves. Thus, the Schwann-spheres enhanced myelin formation and neurite outgrowth compared with the effects of mature Schwann cells *in vitro*.

## Discussion

This is the first report that Schwann-cell precursors/immature Schwann cells, in the form of cultured “Schwann-spheres," can be isolated from adult peripheral nerves. Mature myelinating and non-myelinating cells respond to nerve injury by reverting to a molecular phenotype similar to that of immature Schwann cells, to provide essential support for axonal regrowth [3]. Therefore, we hypothesized that undifferentiated spheres could be obtained from adult injured peripheral nerves. Indeed, here we demonstrated that adult peripheral nerves harvested at specific time points after contusive injury could generate de-differentiated spheres under the floating culture condition with EGF, FGF and fetal bovine serum (FBS). These Schwann-spheres, which exhibited a high self-renewal capacity, consisted of Schwann-cell precursors/immature Schwann cells. Immunocytochemistry and Cre/lox system-mediated lineage tracing analyses showed that the Schwann-spheres originated from myelinating mature Schwann cells, which de-differentiated after peripheral nerve injury. In addition, immunohistochemical and RT-PCR analyses revealed that the Schwann-spheres could differentiate into the Schwann-cell lineage, suggesting that mature Schwann cells de-differentiate into Schwann-cell precursors/immature Schwann cells, but not into neural-crest stem cells, unlike the spheres derived from fetal sciatic nerves or DRGs.

Schwann cells are considered a promising candidate for cellular transplantation therapies to repair the injured central or peripheral nervous system [19], [20], [21]. Previous studies have shown that Schwann cells promote axonal growth, mainly from sensory and propriospinal neurons [22]. Moreover, Schwann cells myelinate the ingrowing axons and re-establish axonal conduction [23]. Although Schwann-cell transplants have shown only limited results, in that few long-tract axons enter and few axons exit the grafts [24], a combination therapy of Schwann cells with neuroprotective agents, molecules that modify the glial scar [25], [26], [27], neurotrophic factors [28], [29], [30], or camp [31], enhances the ingrowth of long-descending axons and the exit of fibers, thereby improving functional recovery. There is a strong current interest in Schwann-cell-based transplantation strategies for the treatment of spinal cord injuries [17]. However, several steps are needed to isolate and obtain highly enriched populations of mature Schwann cells [32], [33]. Moreover, it is difficult to use mature Schwann cells for regenerative medicine because of their low proliferative rate and poor survival when grafted into the injured spinal cord [34], [35].

Recently, Agudo et al. reported the novel and potentially useful properties of an early cell in the Schwann-cell lineage, the Schwann-cell precursor [36]. Unlike mature Schwann cells, transplanted Schwann-cell precursors thrive in the spinal cord, where they survive for a long time. However, Schwann-cell precursors/immature Schwann cells have not been identified in adult tissues, and they have not been prospectively isolated from adult animals, although stem/progenitor cells have been detected in and isolated from fetal peripheral nerves [1].

In the present study, we also demonstrated that the Schwann-spheres derived from injured adult sciatic nerves demonstrated much higher potentials for myelin formation and neurite-growth enhancement than mature Schwann cells isolated from intact sciatic nerves *in vitro*. Skin-derived precursor (SKP)-derived Schwann cells can myelinate axons [37] and enhance locomotor recovery better than naive SKPs, when used as a cell-transplantation source after contusion spinal cord injury [36]. Although the Schwann-spheres differentiated only into the Schwann-cell lineage, and not into the trilineages of neurons, glial cells, and myofibroblasts, they provide a more accessible and potential autologous cell source for transplantation to treat the damaged peripheral or central nervous system, such as occurs in spinal cord injury.

Many investigators have studied stem-cell transplantation therapies for regenerating the central nervous system. Although the transplantation of fetal neural stem/progenitor cells into the injured spinal cord can promote functional recovery in adult mice [38], neonatal rats [39], adult rats [40], and common marmosets [41], the use of fetal tissue-derived stem cells still generates some ethical concerns. To regenerate the central nervous system, multipotent somatic stem cells, which were identified in the adult skin [36], [42] and bone marrow [10], need to differentiate into glial cells prior to transplantation. In addition, hair follicle stem cells [43], [44] promote repair of peripheral nerve injury [45], [46] and spinal cord injury [47], [48]. However, in the results of experiments using neurospheres derived from pluripotent stem cells (embryonic stem cells and induced pluripotent stem cells), transplantation of the gliogenic secondary neurospheres, but not the neurogenic primary neurospheres, promoted axonal growth, remyelination, and angiogenesis after spinal cord injury [49], [50]. Also in such stem cells, to differentiate into glial cells prior to transplantation might be effective for promoting the recovery from spinal cord injury. Interestingly, Widera et al. very recently reported obtaining spheres from the intact sciatic nerve under serum-free medium [51], while we reported observing sphere formation only from the injured nerve under the medium including serum. The former showed that sphere cells were able to differentiate into ectodermal, mesodermal and endodermal cells, while the latter showed that the spheres were able to differentiate only into glial cells. This discrepancy could be partly because of the differences in the culture conditions. In addition, in agreement with the results of others [4], [5], we could not identify nestin-positive cells and spheres in our culture conditions from intact nerves, although we obtained them from injured nerves. Although the spheres that the former reported are more interesting from the biological standpoint, gliogenic spheres such as the spheres in our report might be more effective for the cell transplantation therapies in the same reason. Recently, induced pluripotent stem cells (iPS cells) [52] were recognized as a possible donor source for transplantation therapy, because of their high pluripotency and potential for proliferation. However, a major concern associated with iPS cell-based therapies is tumor formation [53], [54], which is correlated with the persistence of undifferentiated cells that remain after differentiation is induced. In contrast, Schwann-spheres, which contain Schwann-cell precursors/immature Schwann cells, but not neural crest stem cells, mostly differentiate into mature Schwann cells without any specific induction protocol. Taken together, our findings indicate that Schwann-spheres could be a novel candidate for cell-transplantation therapies for the injured central or peripheral nervous system.

## Supporting Information

Figure S1 Trilineage differentiation potential of the spheres derived from the injured adult sciatic nerves of MBP-Cre/Floxed-EGFP mice. EGFP+ spheres derived from the injured adult sciatic nerves differentiated into glial cells (Fig. 3**C**), but not into neurons or myofibroblasts. Glial-cell markers, S100, p75, and P0; neuronal markers, NeuN and Hu; myofibroblast marker, SMA. βIII tubulin and peripherin can label peripheral glia in culture, although they are also known as neuronal markers. Scale bar, 50 µm.
(TIF)
