# Supplementary figures and images for: Schwann-Spheres Derived from Injured Peripheral Nerves in Adult Mice - Their In Vitro Characterization and Therapeutic Potential
Source: PLoS One. 2011 Jun 24;6(6):e21497. doi: 10.1371/journal.pone.0021497 (PMC3123355; doi:10.1371/journal.pone.0021497)

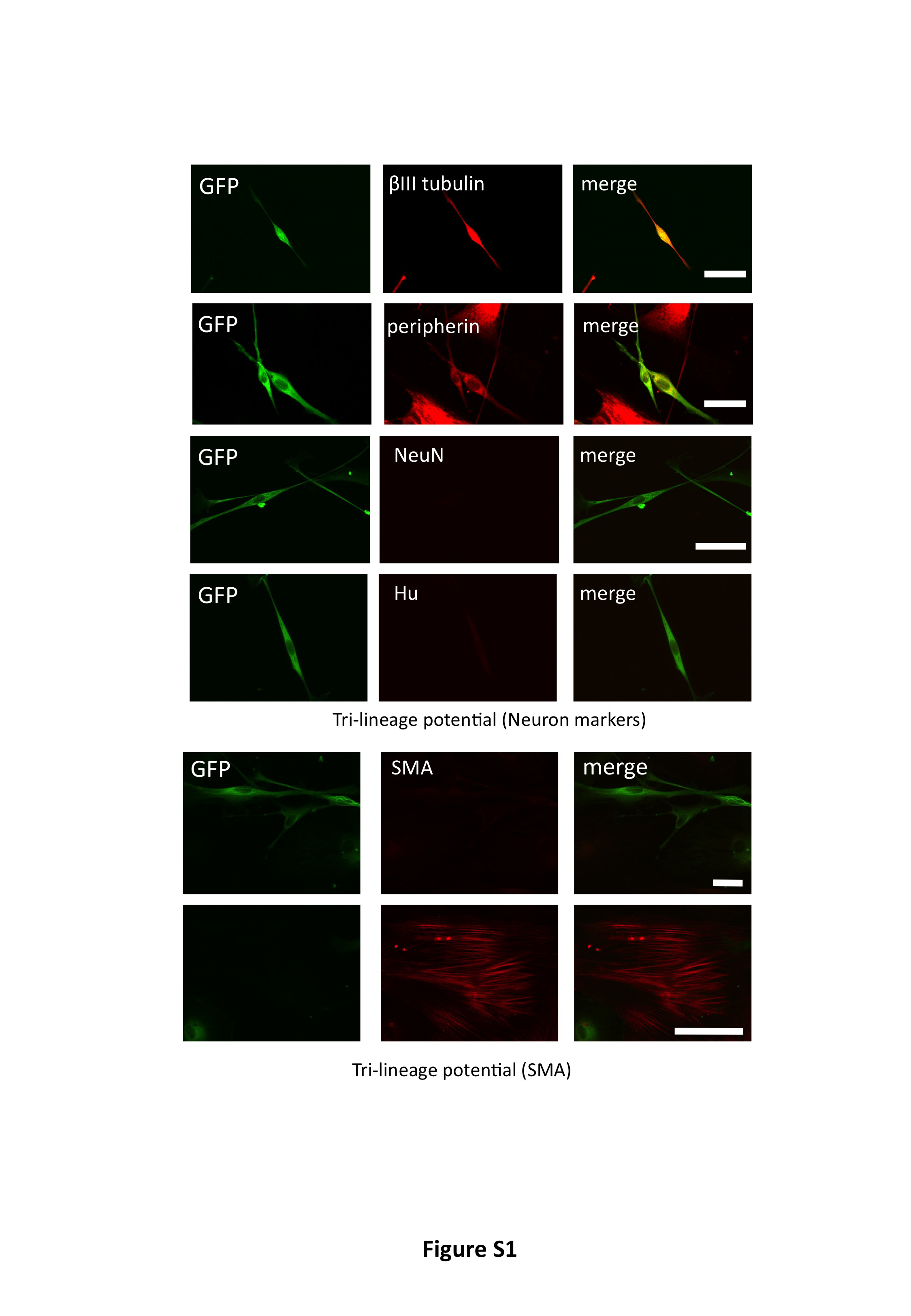

Supplement: Figure S1 — Trilineage differentiation potential of the spheres derived from the injured adult sciatic nerves of MBP-Cre/Floxed-EGFP mice. EGFP+ spheres derived from the injured adult sciatic nerves differentiated into glial cells (Fig. 3C ), but not into neurons or myofibroblasts. Glial-cell markers, S100, p75, and P0; neuronal markers, NeuN and Hu; myofibroblast marker, SMA. βIII tubulin and peripherin can label peripheral glia in culture, although they are also known as neuronal markers. Scale bar, 50 µm. (TIF) [file pone.0021497.s001.tif]
